# Chemomechanical preparation by hand instrumentation and by Mtwo engine-driven rotary files, an ex vivo study

**DOI:** 10.4317/jced.50733

**Published:** 2012-07-01

**Authors:** Károly Krajczár, Zoltán Tigyi, Viktória Papp, Gyula Marada, Jeges Sára, Vilmos Tóth

**Affiliations:** 1 DMD. Clinical Consultant. Dental School, Faculty of Medicine, University of Pécs, Hungary.; 2 MD, PhD Associate Professor. Department of Medical Microbiology and Immunology, Faculty of Medicine, University of Pécs, Hungary.; 3 DMD. Private practice, Nagykanizsa, Hungary.; 4 DMD. Assistant Professor. Dental School, Faculty of Medicine, University of Pécs, Hungary.; 5 BSc, MSc, PhD. Professor. Department of Biostatistics and Health Informatics, Faculty of Health Sciences, University of Pécs, Hungary.; 6 DMD, PhD. Professor. Dental School, Faculty of Medicine, University of Pécs, Hungary.

## Abstract

Objective: To compare the disinfecting efficacy of the sodium hypochlorite irrigation by root canal preparation with stainless steel hand files, taper 0.02 and nickel-titanium Mtwo files with taper 0.04-0.06. Study Design: 40 extracted human teeth were sterilized, and then inoculated with Enterococcus faecalis (ATCC 29212). After 6 day incubation time the root canals were prepared by hand with K-files (n=20) and by engine-driven Mtwo files (VDW, Munich, Germany) (n=20). Irrigation was carried out with 2.5% NaOCl in both cases. Samples were taken and determined in colony forming units (CFU) from the root canals before and after the preparation with instruments #25 and #35. Results: Significant reduction in bacterial count was determined after filing at both groups. The number of bacteria kept on decreasing with the extension of apical preparation diameter. There was no significant difference between the preparation sizes in the bacterial counts after hand or engine-driven instrumentation at the same apical size. Statistical analysis was carried out with Mann-Whitney test, paired t-test and independent sample t-test. Conclusions: Significant reduction in CFU was achieved after the root canal preparation completed with 2.5% NaOCl irrigation, both with stainless steel hand or nickel-titanium rotary files. The root canal remained slightly infected after chemo mechanical preparation in both groups.

** Key words:**Chemomechanical preparation, root canal disinfection, nickel-titanium, conicity, greater taper, apical size.

## Introduction

Microorganisms play an essential role in the initiation, progression and persistence of apical periodontitis (1,2). Therefore, endodontic therapy aims to eliminate bacteria from the infected root canal and prevent reinfection. Cleaning and shaping the root canal reduces the number of bacteria (3,4). But in the hard-to-reach areas and in the dentinal tubules of the root canal system microorganisms putatively will be left behind contrary to the instrumentation and irrigation (5-12).Bacteria are able to penetrate into dentinal tubules up to 400 ?m (13-17). Instrumentation opens a way for disinfectant solution to reach most of the enclosed infected parts of root canals (18), but the extension of shaping is restricted by the thickness of the dentine wall and threatens with the possibility of perforation (10,11,19). There are data in the literature concerning the necessary file size of the hand instruments recommended to achieve satisfactory disinfection (11,20,21,22), but no similar conventions are accepted concerning the rotary files.

The aim of the present study was to compare the disinfection effectiveness of the chemomechanical instrumentation of rotary nickel-titanium files and hand files at two apical diameters. The number of bacteria left behind was detected at two different apical sizes, recommended for root canal filling (minimal apical size = #25) (3) and at a greater one (#35) (23).

## Material and Methods

Forty-five extracted human teeth with single unprepared root canals were selected. Apical areas were examined at 3 times magnification for signs of the lack of immaturity and external resorption.

The anatomical crown was removed with a diamond disc at the level of cementoenamel junction to expose the pulp chamber. A #15 K-file (VDW, Munich, Germany) was negotiated into the root canal until it appeared at the apical foramen to determine the length of the canal. The working length was considered one mm shorter then the previously determined canal length. The outer surfaces of the roots were covered with two layers of nail-polish (5). With the help of cylindrical shaped templates, each root was mounted vertically into a resin (Vertex Self Curing, Vertex – Dental, Zeist, The Netherlands) Block, 30 mm in height and 20 mm in diameter.

The coronal 2-3 mm part of the roots was left out from the plastic blocks. After setting the acrylate blocks, root canals were irrigated with 1 ml 17 % EDTA solution for 1 minute to open the dentine tubules (13) and than irrigated with 2 ml physiologic saline solution to wash out the EDTA. Specimens covered with aluminum foil and autoclaved for 15 minutes at 121°C at a pressure of 2 bars.

All further preparations were performed under sterile conditions with sterile instruments in a laminar airflow cabinet. Samples were divided at random into three groups.

 Group one, (n=5), served for control to check the sterilization process. Group two (n=20) received conventional stainless steel hand instrumentation (VDW, Munich, Germany). Group three (n=20) nickel titanium rotary Mtwo files instrumentation (VDW, Munich, Germany) was performed.

The sterilized root canals of group 1 were covered off the aluminum foil and filled up with physiologic saline solution. Gentle filing with #15 K-file was performed to reach the working length, and then three consecutive samples were taken with #15 sterile paper points (VDW, Munich, Germany). Brain-heart infusion broth was inoculated with the samples and incubated at 37°C for 48 hours. Then the samples were spread on Müller-Hinton agar plates and incubated for another 24 hours. Colony forming unit (CFU) counts of samples were determined (7).

In the case of groups 2 and 3 root canals were inoculated with a suspension containing Enterococcus faecalis (ATCC 29212) in glucose-yeast medium (7,13,17,24). Preliminary experiments were performed to determine the optical density measured at 600 nm wavelength of the bacterial cell suspension and its corresponding bacterial cell count.

According to this studies 0,2 value of the optical density at ?=600nm corresponded to 2,7 x 106 CFU/ml. The suspension was delivered into the canal with a 2 ml sterile syringe and 27G needle. The fluid was agitated with gentle back-and-forth movement of #15 K-file in the canal, to allow the equal distribution of the bacterial suspension and release air bubbles. The penetration of the medium and bacteria into the dentinal tubules was en-hanced by ultrasonic treatment of the blocks, placing them into cup horn ultrasonic head filled with mixture of water and ice (Cole Parmer High Intensity Ultrasonic Processor, Microprocessor Controlled 600 Watt Model, Cole Parmer, Vernon Hills, Illinois); power 20 W, 8 min, impulse mode: 9.9 sec on, 5 sec off). Preliminary experiments proved this kind of ultrasonic treatment safe concerning the bacterial cell viability. Our preliminary experimental data showed that the ultrasonic treatment applying the above parameters did not affect the bacterial cell viability. Blocks were wrapped in sterile aluminum foil and incubated in an airtight closed wet chamber at 37°C for 6 days. Canals were refilled with Glucose-yeast medium every 2. day (5).

Root canal instrumentation of groups 2 and 3 was carried out by the same person and assistant, on the 6th day after the inoculation. Root canals were irrigated before mechanical preparation with 1ml physiologic saline solution (2 ml syringes and 27G needles), to remove the unattached bacteria. In group 2 mechanical root canal preparation was performed to the whole working length with series of K-files (#15, #20, #25, #30, #35, #40, 0.02 taper) with circumferential filing movement. The instrumentation began with #15 K-file and at the end of the preparation the file was taken with the debris on it into a test tube containing 1 ml physiologic saline solution. After that 3 sterile #15 paper points were put into the root canal consecutively to collect the residual rinsing solution and debris from the root canal. They were added to the test tube (5). This sampling (S1) defined the extent of the initial infection of the root canal wall. Then the instrumentation was continued with #20 and #25 K-file and the canal was irrigated with 1.5 ml 2.5% NaOCl after every instrument size. The second bacterial sampling (S2) was intended to determine the degree of the remaining infection after a #25 apical size file in-strumentation. The antibacterial effect of the residual disinfectant was neutralized with 1.5 ml 5% sodium-thiosulphate irrigation, followed by 1 ml physiologic saline solution. The preparation was continued with #30 K-file. The file and the debris carried on it were put into a test tube containing 1 ml of physiologic saline solution. The residual fluid from the canal was soaked up with three consecutive sterile #30 paper points and was added to the solution. Then the canal irrigation and neutralization steps were repeated as mentioned above. The third sample group (S3) was collected alike S2 but with #40 K-files and #40 sterile paper points. S3 was meant to determine the extent of the root canal infection left after the apical preparation with the file size #35.

Endo IT Professional (VDW, Munich, Germany) endodontic motor and #15/0.05, #20/0.06, #25/0.06, #30/0.05, #35/0.04 and #40/0.04 size Mtwo files were used for group 3 preparation. Every instrument was introduced to the working length. Canals were irrigated with 1 ml physiologic saline solution before being prepared with #15/0.05 file. Three samples were taken with #15/0.05, #30/0.05 and #40/0.04 files and with paper points #15, #30 and #40 three pieces of each, respectively. Canal irrigation and the neutralization process of the disinfectant solution was identical to group 2. This sampling method of the CFU count allowed to determine the bacterial cell number left in the canals after the preparation with Mtwo files #25/0.06 (S2) and #35/0.04 (S3).

Test tubes containing S1 samples of groups 2 and 3 were vortexed at 40 1/sec for 5 sec (Velp Scientifica, Mila-no, Italy) (7,17), and a ten- and hundredfold dilutions were made in parallel on microtitration plate (Sarstedt AG & Co., Nümbrecht, Germany). 10?l of sample was taken out from each dilution and germ counts were determined by means of the droplet method (25) on Müller-Hinton medium. S2 and S3 samples were treated in the same way, but with no dilution, while previous studies showed that lower numbers of bacteria could be expected. Colony forming units were counted in front of a black background on tenfold magnification after incubation at 37°C for 24 hours. The mean of the six CFU values gained from one dilution was calculated in each sample. Statistical analysis was carried out with Mann-Whitney test, paired t-test and independent sample t-test.

## Results

In the culture harvested from the first sample (control group) bacterium was not found, so the sterilization of the samples proved to be appropriate.

Results from group 2 and 3 were summarized in  Table 1. The initial values (S1) of groups 2 and 3 didn’t differ significantly according to the Mann-Whitney test. The CFU number of sample No. 9 in group 2 increased sharply during the treatment was supposed to be contaminated, so it was excluded from the study.

**Table 1:**
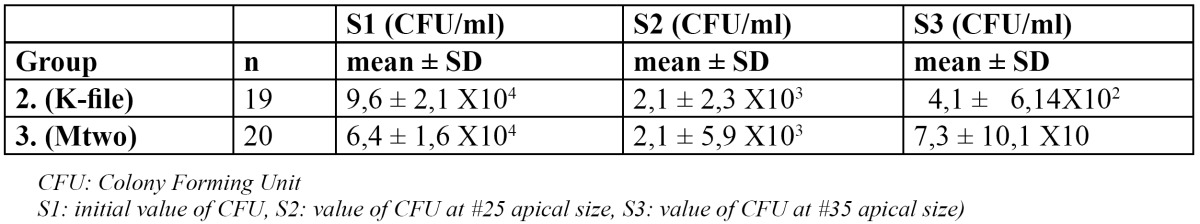
Enterococcus faecalis CFU/ml count at the initial (S1), at #25 (S2) and at #35 (S3) apical preparation size.

**Figure 1:**
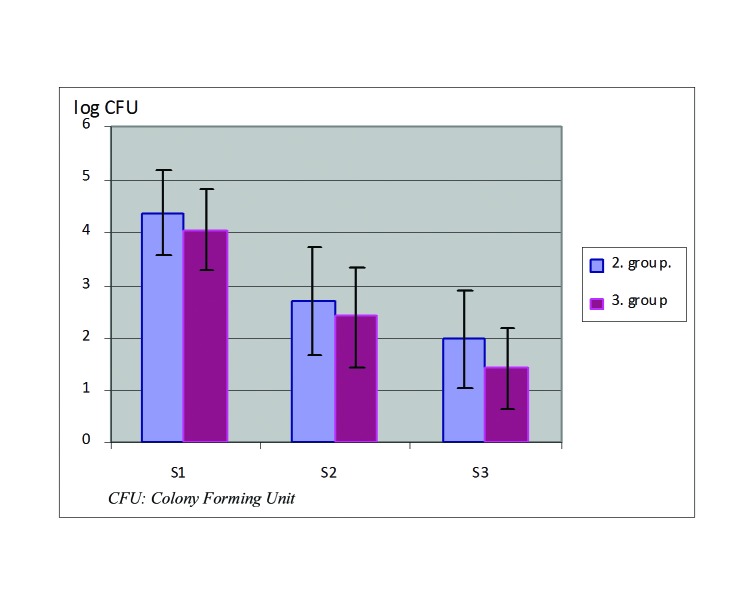
The decrease of logarythm of CFU/ml at group 2 and 3 during preparation.

During the consecutive steps of root canal treatment, either with the conventional method or rotary files, the bacterial count decreased. The initial percentage of the bacterial count (S1=100%) was fallen down to 2.06% and 0.36% in group 2 and to 3.2% and 0.11% in group 3 at the second (S2) and third (S3) samples respectively (Fig. 1).

The CFU counts of consecutive samplings were compared with paired sample test in both study groups. The logarithm of the difference of numbers was taken. There were significant decreases in the bacterial counts among the samples (S1, S2 and S3) in both groups. ( Table 2).

**Table 2:**
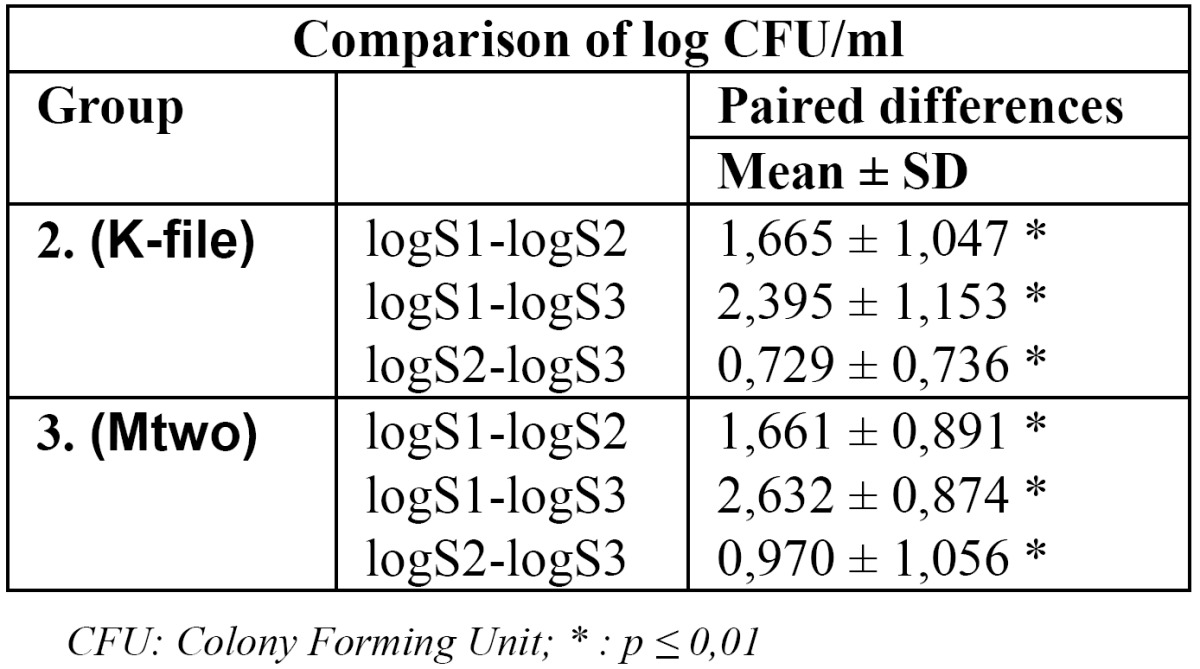
Comparison of log CFU/ml at S1, S2 and S3 in group 2 and 3.

Data of the two study groups were analyzed with independent samples test. No significant difference was found between the two preparation methods concerning the reduction of the bacterial counts at the same apical sizes ( Table 3).

**Table 3:**
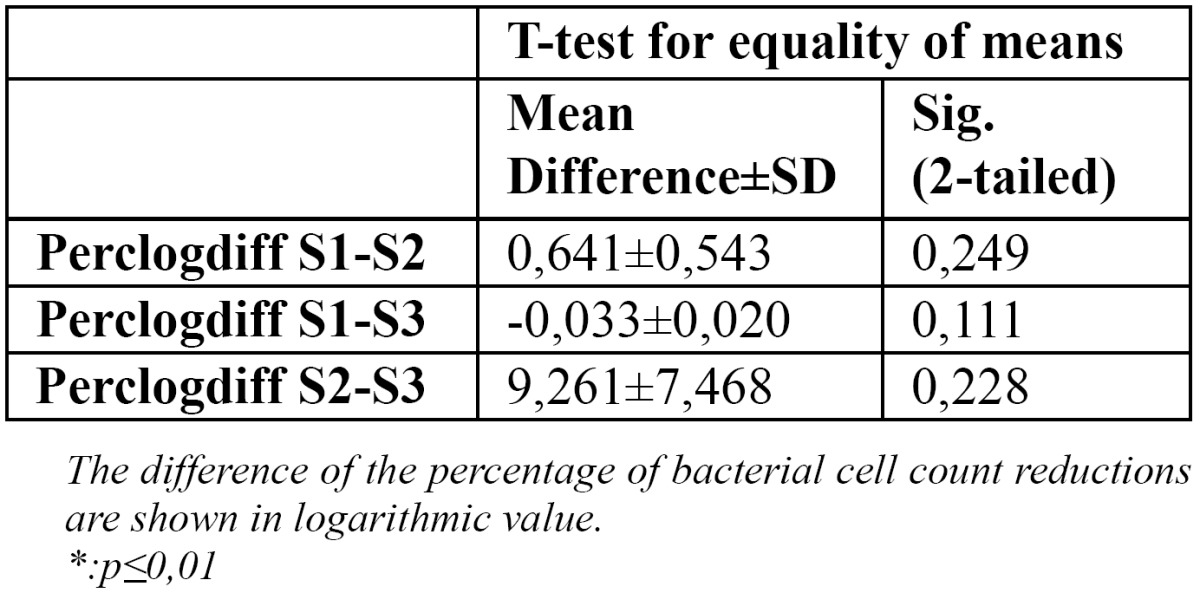
Comparison of the bacterial cell counts reduction of the two preparation methods at the same diameters.

A significant bacterial count number decrease was found parallel to the increasing apical preparation size which seemed to be independent from the preparation method.

## Discussion

Enterococcus faecalis facultative anaerobic bacteria are able to induce long standing apical periodontitis. Numerous previous experiments were used to build a model for studying the cleaning and disinfection efficacy of several endodontic methods in vitro (7,13,17). In our study on extracted teeth the inoculation of the root canal system and the dentin tubules were carried out with *E. faecalis* and was facilitated by gentle ultrasonic agitation. In other studies, root canals were filled first with sterile medium followed with the bacterial inoculation (13). In our model the root canals were filled with *E. feacalis* containing medium to ensure more even bacterial distribution even in the deeper areas in the root canal system. Due to this method no significant difference was found in the bacterial counts after the established bacterial inoculation (S1) between the study groups 2 and 3.

Root canal preparation was performed with chemo mechanical method in both experimental groups. In our study the mutual effect of mechanical preparation and disinfectant solution was examined. In our chemo mechanical root canal preparation model the combined effect of the disinfection with 3.5% NaOCl and mechanical instrumentation was rigorously utilized and their combined effect detected. The impact of the method of mechanical instrumentation i.e. hand instruments or rotary files, was compared at the same apical size for the purpose to adopt the well established conventions, which apical instrumentations are acceptable, developed with hand instruments.

After preparation with #25 and #35 files sampling with the next instrument size were carried out to collect bacteria left from the root canal wall and from the contaminated pulpal dentin tubules as well. Root canal preparation to apical size #25 is the minimal diameter advised to reach for root canal filling (3). Further preparation the next apical size showed an additional significant bacterial count reduction ( Table 2) irrespectively from the preparation method. Our results are in good accordance with previous studies (7,10,11,12,19,20,26). According to our findings we suppose that further reduction of bacterial counts may be achieved with larger preparation sizes due to the further removal of infected dentin and with the exploration of the anatomic irregularities. Root canals became wider either in the coronal and in the apical regions at both methods, so a more intensive irrigation effect could be achieved (11,27,28). In our study, with accordance to other previous ones, the less taper of hand instruments with filing movement proved not to be less effective as rotary instruments with greater tapers at the same apical size (5,10). The rate of the bacterial count reduction in this study may be influenced by the neutralization of the NaOCl solution, but it was essential for the reliability of samplings (5,29).

Dalton et al. (10) reached significant bacterial reduction with mechanical preparation and irrigation with physiologic saline solution, but could not reach a germ-free root canal system. Chuste-Guillot et al. (5) compared the disinfecting efficacy of the preparation with nickel-titanium and stainless steel hand instruments together with 3.5% NaOCl irrigation. Significant bacterial reduction was detected, but the root dentin remained still contaminated. Coronal conicity was considered to be the most important factor in germ number reduction. Antimicrobial irrigant was supposed to have a better effect in a steeply tapered canal.

Machado et al. (30) compared the root canal disinfection efficacy of ProTaper and Mtwo systems contaminated with *E. faecalis*. ProTaper and Mtwo are rotary systems that have different characteristics with respect to their conicity. They concluded that the germ number reduction of both systems is similar using sterile distillated water irrigation. It was also concluded that significant germ number reduction could be achieved by increasing the size of preparation at both system.

According our results the root canal preparation with Mtwo engine driven nickel titanium instruments with greater taper and with hand instruments with conventional (0,02) taper provided similar disinfection efficacy at the same apical size. This result could be considered excellent regarding the antibacterial effectiveness of both methods concerning the initial great measure of bacterial contamination. But it has to be accepted that 1-2% of bacteria are not accessible for the mutual effect of mechanical preparation and disinfecting irrigants during root canal treatment (7,10).
